# Incidence rate and implications of splenic ischemia during sleeve gastrectomy and its related procedure

**DOI:** 10.1097/MD.0000000000037276

**Published:** 2024-03-01

**Authors:** Jinrong Huang, Chenglong Liao, Weiwei Zhou, Hancheng Li

**Affiliations:** a Department of Gastrointestinal Surgery, the Ningde Municipal Hospital of Ningde Normal University, Ningde Fujian, P. R. China.

**Keywords:** bariatric surgery, complications; sleeve gastrectomy, splenic infarction, splenic ischemia

## Abstract

Splenic ischemia (SI) is a common finding during sleeve gastrectomy (SG) procedures; however, reports are still lacking. In this study, we retrospectively analyzed our SG patients to understand better the incidence rate and implications of SI. Patients’ data from the beginning of the year 2021 until December 2022 that underwent bariatric surgery at our university hospital were retrospectively analyzed. Patient surgery video was reviewed by all the authors to investigate the incidence of SI. Thereafter, the corresponding patient age, height, weight, BMI, and their postoperative day 1 (POD1) temperature and blood routine test results (patients were routinely discharged at POD2) were collected and analyzed. 204 patients were included in this study. The mean age and preoperative BMI were 31.7 ± 7.4 years old and 38.8 ± 5.6 kg/m^2^, respectively. SI was observed in 18 cases (8.8%). 30-day readmission rate was seen in 3 patients (1.5%, all without SI during the primary surgery). There was no statistical difference with regard to the POD1 temperature and blood test results between the patients with and without SI. The incidence of SI during sleeve gastrectomy-related procedures is a common finding in our study. We did not observe significant differences postoperatively between the patients with and without SI before discharge. Further study is needed to understand the mechanism for the incidence of SI during SG.

## 1. Introduction

Sleeve gastrectomy (SG) is one of the most common bariatric procedures worldwide.^[1]^ Although relatively a simpler procedure, complications do occur following SG.^[2]^ Splenic ischemia (SI) following SG is one of the possible complications, however, its significance of is still unclear.

Most studies relating to SI during SG were reported as case reports,^[3,4]^ while one study reported a rate of 4.1% of SI during SG.^[5]^ The reported incidence of SI during SG procedure could be underestimated; however, it is difficult to conclude with such limited reports available. Most studies suggested that SI after SG might have little to no clinical relevance, but sequalae into abscess formation have been reported.^[6–9]^ Furthermore, it is uncertain whether SI during SG is preventable, thus, improving the quality of the surgery.

In this study, we retrospectively reviewed the data and videos of our bariatric patients, to compare the postoperative day 1 (POD1) temperature and the routine blood test results between patients with and without SI during SG. As most of our patients were discharged at approximately 48 hours following surgery, this study was aimed to better understand whether the patients with SI would need for longer postoperative observation. Furthermore, we further observed and investigate the most probable cause of the incidence of SI during the SG procedure.

## 2. Materials and methods

This study was approved by the Ethics Committee and institutional review of *our university hospital* and was compliant with the Helsinki Declaration. All patient received detailed communications for the understanding relating to the laparoscopic sleeve gastrectomy procedure and had signed the informed consent before the surgery. This is a retrospective study.

### 2.1. Data selection

Patients’ data from the beginning of the year 2021 until December 2022 that underwent bariatric surgery at *our university hospital* were retrospectively analyzed; all patients were of ethnic Chinese. The inclusion criteria were as follows: underwent SG or SG-related procedure as a primary bariatric surgery; without previous bariatric or abdominal surgeries, nor having cardiovascular, neurological, or malignant underlying diseases.

Patient age, height, weight, BMI, and their respective POD1 temperature, heart rate and blood routine test results (taken at approximately 7 am) were collected and analyzed. Patient video was reviewed by all the authors to investigate the incidence of SI.

### 2.2. Sleeve gastrectomy and with transit bipartition procedure

Our overall SG procedure was described previously.^[10]^ Briefly, omental dissection was performed until the angle of His and the left crus was visualized, allowing complete mobilization of the fundus; 36 French bougie tube was used; entire staple line was reinforced using Covidien 3-0 V-Loc suture to bury the staple line. Three-port SG was performed for patients with BMI < 40 kg/m^2^, 4-port for BMI between 40 and 49 kg/m^2^ and 4 or 5-port for BMI ≥ 50 kg/m^2^.

For the SG with transit bipartition (SG-TB) procedure, the SG procedure was as mentioned above, with additional gastrointestinal anastomosis at the gastric antrum (intestine was measured 260 cm from the ileocecal valve) and intestinal-intestinal anastomosis (measured 30 cm away from the gastrointestinal anastomosis). Five-port was used for SG-TB procedure.

### 2.3. Statistical analysis

All data are presented as mean ± SD. Student *t* test was used to compare the differences between mean. All tests were 2-tailed and are statistically significant when *P* < .05.

## 3. Results

215 patients performed SG-related procedures were recorded. 11 patients were excluded (10 due to unclear videos and 1 was a revisional surgery), resulting in 204 patients included in this study (173 SG and 31 SG-TB). The mean age was 31.7 ± 7.4 years old. The mean preoperative BMI was 38.8 ± 5.6 kg/m^2^. No conversion was needed for all the surgery, and no mortality was recorded. 30-day readmission rate was 1.5% (1 SG and 2 SG-TB patients, all without SI during the primary surgery). One SG patient was re-admitted due to suspected portal vein thrombosis (the patient had cirrhosis preoperatively) and was sent to intervention radiologist for further treatment. One SG-TB patient was re-admitted due to possible leakage at the gastrointestinal anastomosis (infected abdomen but leak point was not located). The other SG-TB patient was re-admitted due to abdominal pain, minor bloody vomitous and without fever; this patient was treated conservatively and discharged.

**Figure 1.**
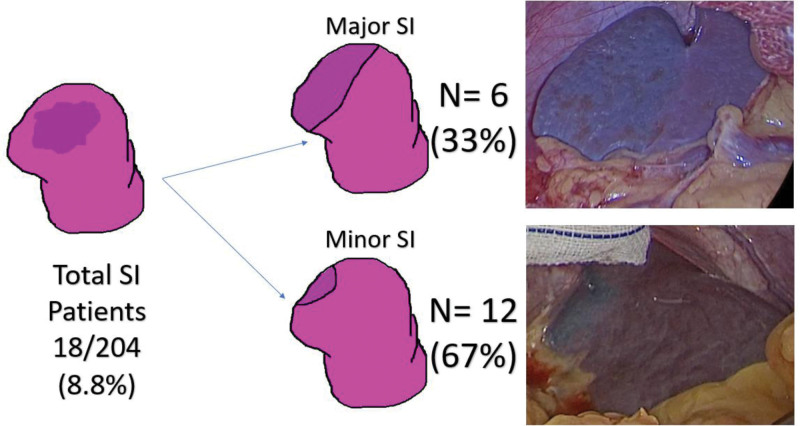
Prevalence of major and minor splenic ischemia (SI).

SI, interpreted as demarcation/discoloration of the spleen, was observed in 18 cases (8.8%) (Fig. 1.). Major SI (large upper pole demarcation) was observed in 6 patients (33.3%), while a minor SI (localized upper pole demarcation) was observed in 12 patients (66.7%). Observations were made only through the review of the videos.

**Figure 2.**
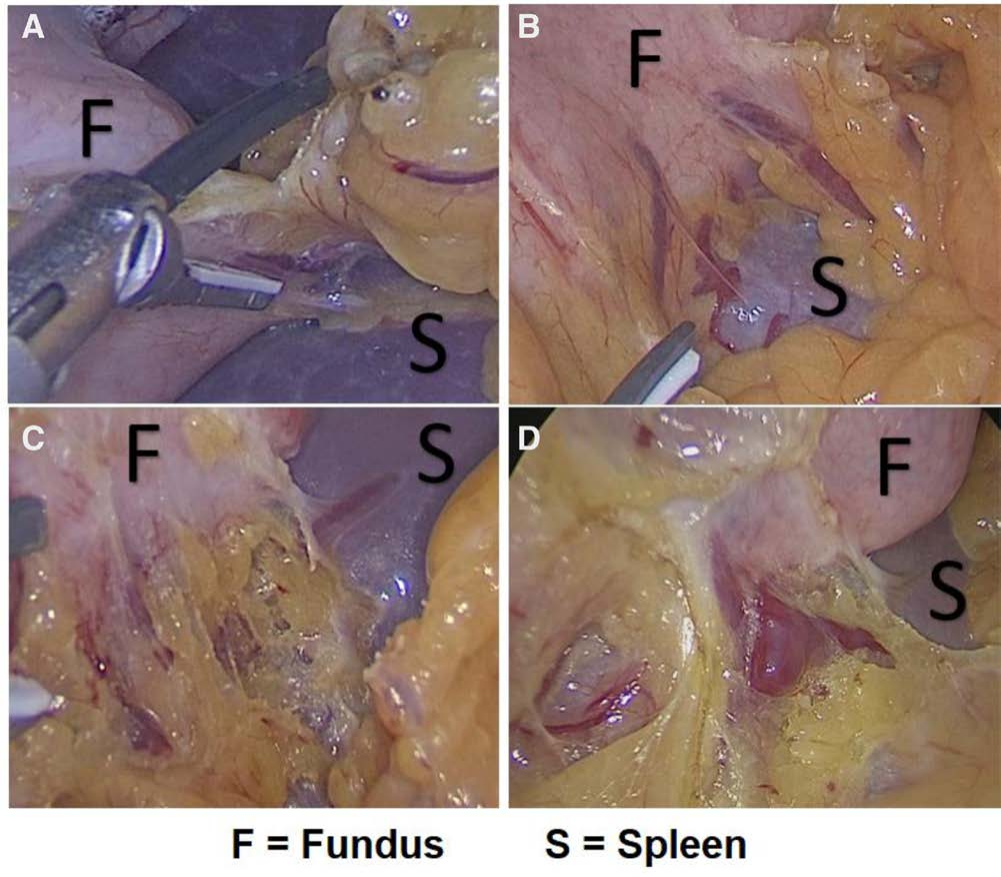
Demonstrate the anatomical variations of the short gastric vessels seen in patients with splenic ischemia. F = fundus, S = spleen.

In all SI cases, we observed a very short fundus-omentum-spleen distance. Furthermore, in patients with SI, we frequently observed the short gastric vessels of the greater curvature leading directly to the spleen, and these vessels were consequently ligated and dissected as to further mobilize the fundus (Fig. 2.).

In terms of the POD1 temperature, the mean temperature between the SI and no-SI patients was 36.9 ± 0.4ºC and 36.8 ± 0.4ºC, respectively (Table 1). The mean postoperative hospital stay between the SI and no-SI patients was 2.65 ± 0.93 days and 2.47 ± 0.80 days, respectively. There was no statistical difference with regard to the POD1 temperature and the mean postoperative hospital stay between the 2 groups.

**Table 1:** Data analysis for the patients with and without splenic ischemia.

Variables	Splenic infarction N = 18	Normal N = 186	*P* value
Gender (F/M)	16/2	144/42	-
Age, yr	33.1 ± 7.9	31.6 ± 7.3	.4515
Weight, kg	101.0 ± 16.7	109.6 ± 22.6	.1222
Height, m	1.65 ± 0.06	1.67 ± 0.08	.0547
BMI, kg/m^2^	37.1 ± 5.9	39.0 ± 5.6	.1887
Temperature, ºC	36.9 ± 0.4	36.8 ± 0.4	.0630
Heart rate	81.7 ± 14.5	81.5 ± 12.0	.9724
WBC, *10^9/L	10.5 ± 2.1	11.6 ± 2.8	.0531
NEU%	81.8 ± 4.4	80.2 ± 5.5	.1724
LYM%	13.5 ± 4.2	13.7 ± 4.4	.8496
HB, g/L	120.8 ± 10.4	126.2 ± 13.3	.0515
Platelet, *10^9/L	238.9 ± 41.4	246.4 ± 56.5	.4876
CRP, mg/L	31.4 ± 26.1	25.0 ± 21.5	.3264

Data are shown as mean ± S.D. *P* value is significant at <.05.

BMI = body mass index, CRP = c-reactive protein, HB = hemoglobin, LYM% = percentage of lymphocyte, NEU% = percentage of neutrophil, WBC = white blood cell.

In terms of the POD1 routine blood test results (SI vs no-SI): WBC (white blood cell) 10.5 ± 2.1 versus 11.6 ± 2.8 *10^9/L; NEU% (percentage of neutrophil) 81.8 ± 4.4 versus 80.2 ± 5.5%; LYM% (percentage of lymphocyte) 13.5 ± 4.2 versus 13.7 ± 4.4%; Hemoglobin 120.8 ± 14.3 versus 126.2 ± 13.3 g/L; Platelet 238.9 ± 41.4 versus 246.4 ± 56.5 *10^9/L; CRP (C-reactive protein) 31.4 ± 26.1 versus 25.0 ± 21.5 mg/L. No statistical significance was observed in all the parameters.

## 4. Discussion

In this study including 204 SG-related patients, 18 cases (8.8%) of SI were observed. There were no clinical symptoms such as abdominal pain and high fever observed in all SI patients before discharged. Three patients (1.5%) were re-admitted within 30 days postoperatively due to complications and were treated consequently, these patients were all without SI. Furthermore, there were no significant differences in terms of the POD1 routine blood test results between the patients with and without SI. Our study suggests that the incidence of SI in SG-related procedure was common, however, we did not observe the significance of SI in terms of the physiological *harm* to the patients. Furthermore, the SI cases observed in our study were highly likely related to the short fundus-omentum-spleen distance (in addition to the greater curvature short gastric vessels leading directly to the spleen).

Our incidence of SI following SG-related procedures (8.8%) was higher than that of reported by Stamou et al (4.1%, total n = 287) and Nassour et al (5%, *total number of patients was not mentioned*).^[5,8]^ However, due to the lack of reports regarding the incidence of SI after bariatric surgery, we cannot conclude the findings of our study with the others. We are yet able to judge whether our incidence of SI was too high or whether the other authors findings were too low. Therefore, we propose that all future studies report the incidence of SI when reporting their SG-related procedures.

Although SI following SG might have little to no clinical relevance, several authors have reported the incidence of splenic abscess that could have been related to SI.^[6–9]^ Unlike a simple SI, the sequelae of splenic abscess might result in abdominal pain and fever and might need interventions. Furthermore, the symptoms of splenic abscess following SG are similar to the sleeve-leak, thus, complicating the diagnosis process.

If the sequelae of splenic abscess are to be related to SI *(though we did not find any in our study*), thus, preventing SI during the procedure could be deemed important. However, whether we could prevent SI during the procedure is still unclear. We observed several impasses in preventing SI, such as the short distance between the fundus and the spleen, and the short gastric vessels connecting the greater curvature directly to the spleen. Although we cannot conclude that these are the main reasons for the SI occurrence in SG procedure, we strongly believed that they are the likely culprit. Better studies are needed to understand the relation of SI and bariatric surgery, why these SI occurs? Why did it not occur in most patients? And whether it is preventable.

Our study suggests that the incidence of SI during SG-related procedures did not result in additional harmful physiological effect to the patients. There were no significant differences between the patients with and without SI postoperatively, before discharge. We did not observe further side effects related to the SI in all the patients as judged by the 30-day readmission rate. However, although rare, careful to the SI patients are still recommended as splenic abscess sequelae have been reported by others.^[6–9]^

Our study is limited by the low number of patients and postoperative CT scan was not performed. With postoperative CT scan, we will be able to understand the progression of SI. However, without clear indications such as fever and/or abdominal pain, CT scan is not routinely performed preoperative and postoperatively. Further studies will be needed, possibly multi-centered studies might be better, to judge as well whether the incidence rate of SI is related to the surgeons performing the surgery. Furthermore, the postoperative blood test in this study was limited to only the routine complete blood count and CRP.

## 5. Conclusion

The incidence of SI during sleeve gastrectomy-related procedures is a common finding in our study. We did not observe significant differences postoperatively between the patients with and without SI before discharge. Thus, our current study suggests that the incidence of SI is without clinical relevance. Furthermore, SI might not be easily prevented due to anatomical differences in each patient.

## Author contributions

**Conceptualization:** Jinrong Huang, Hancheng Li.

**Data curation:** Jinrong Huang, Chenglong Liao, Weiwei Zhou, Hancheng Li.

**Formal analysis:** Jinrong Huang, Chenglong Liao, Hancheng Li.

**Funding acquisition:** Jinrong Huang, Hancheng Li.

**Investigation:** Jinrong Huang, Chenglong Liao, Hancheng Li.

**Methodology:** Jinrong Huang, Chenglong Liao, Weiwei Zhou.

**Project Administration:** Jinrong Huang.

**Resources:** Jinrong Huang, Hancheng Li.

**Software:** Jinrong Huang.

**Supervision:** Jinrong Huang.

**Validation:** Jinrong Huang.

**Visualization:** Jinrong Huang.

**Writing – original draft:** Jinrong Huang.

**Writing – review & editing:** Jinrong Huang.
